# In this digital age, how easily accessible is pharmacist vaccination information? The case of New Zealand

**DOI:** 10.1016/j.rcsop.2021.100033

**Published:** 2021-06-12

**Authors:** Georgia Bell, Shekiba Ikhtiari, Ashley Johns, Devin Teoh, Shane Webber, Patti Napier, Mudassir Anwar

**Affiliations:** a School of Pharmacy, University of Otago, 18 Frederick Street, Dunedin 9010, New Zealand; b Southern District Health Board, Dunedin, New Zealand

**Keywords:** Vaccination, Pharmacist vaccination, Promotion, Online platforms, New Zealand

## Abstract

**Background:**

Pharmacist-led vaccination that has the potential to ease the vaccination burden from general practitioners, is comparatively a newer service in New Zealand. However, to reap the maximum benefits out of this service, a consistent and effective promotion approach using various online platforms is indispensable.

**Objective:**

To identify what online information the general public can find about which pharmacies across NZ provide vaccination services.

**Methods:**

Every pharmacy in NZ was reviewed online to determine what vaccination information they advertised, then a sample of pharmacies were randomly selected from six District Health Boards (DHBs) to be called and confirm if the information they stated online was accurate.

**Results:**

Whilst the majority (more than 70%) of pharmacies did provide information about their services online, only 31% of the pharmacies had vaccination information on their websites, 20% on Healthpoint, and 13% had the information on social media. The telephonic survey revealed various information discrepancies in more than a quarter of the sample.

**Conclusions:**

A lack of online presence across multiple pharmacies is a pressing issue. Also, currently, NZ pharmacies do not have a very high online presence advertising vaccination services. Improving the amount and quality of this information is pertinent at this time as when COVID-19 vaccination drive may commence anytime, and the pharmacy sector will be well placed to conduct vaccinations on a large scale.

## 1 Introduction

Vaccination has long been endorsed as crucial in providing immunological protection against a variety of infectious diseases. Groups such as the World Health Organisation (WHO) promote immunisation as an effective method to prevent disease.^1^ The availability of vaccines in varying locations is one way to promote and make them more accessible to the public. Increased utilisation of pharmacists in all stages of the immunisation programme can lead to better awareness of vaccines.^2^ It could also ease the vaccination burden from General Practitioners (GPs), and be effectively carried out across a large population of people.^3^

One of the immunisation aims of the New Zealand (NZ) government is to achieve high levels of ‘Herd Immunity’; where significant vaccination levels interrupt the chain of transmission.^4^ This threshold must be surpassed to best protect vulnerable populations from becoming infected (e.g. Herd Immunity Threshold for Pertussis is 80–94%).^4^ Herd immunity is critical for the vulnerable in the New Zealand population. The greater the accessibility of vaccines, the less likely it is that widespread disease will occur.^5^

To achieve such high levels of vaccine coverage, the concept of technological integration and promotion should be considered. Through consistent, accurate, and accessible online information regarding the ability of pharmacies to vaccinate, the vaccination burden could be eased from GPs, and effectively carried out across a large population of people.^3^

Vaccination campaigns have been developed as opportunities to educate local communities about preventable diseases and which vaccines are available to them.^6^ One such campaign is the annual national influenza promotional campaign, which aims to vaccinate 75% of the population 65 years or older and other ‘high-risk groups’. They also aim to vaccinate 80% of healthcare workers against influenza.7., 8., 9.

Despite the implementation of vaccination campaigns around the country, there are still groups of individuals who are hesitant or refuse to receive vaccines.^10^ Vaccination rates in NZ have declined since 2016. As a result, preventable diseases such as measles have flared up and spread, reaching 100 cases across the country in 2019.^11^^,^^12^

During the midst of the 2020 COVID-19 pandemic, District Health Boards (DHB) such as the Taranaki DHB have pushed for an increase in the uptake of vaccines; particularly the annual influenza vaccine.^13^ Even though this vaccine does not convey protection to patients from COVID-19, the intention is to minimise the burden that influenza has on the health system and reduce the demand for hospital beds.^14^

Pharmacists vaccinating in NZ has evolved since the accreditation of 22 pharmacists in 2011, following the reclassification of the influenza vaccine.^15^ The number of registered pharmacist vaccinators has grown to around 1000 (September 2019) and is predicted to increase, with changes at this time allowing for intern pharmacists to complete training for influenza vaccine administration.^16^^,^^17^ From a small trial of the influenza vaccine, the scheme now includes oral *Vibrio cholerae* and enterotoxigenic *Escherichia coli* vaccines, Diphtheria/tetanus/pertussis (Tdap), Meningococcal and Shingles vaccines.^18^ As a result of the 2019 measles outbreak, the Measles, Mumps, and Rubella (MMR) vaccine was also temporarily reclassified, illustrating how changes can be made in response to increased need. All pharmacist vaccinators must pass an approved Vaccinator Training Course (VTC) and meet the necessary standards.^19^

In New Zealand, each DHB has the responsibility of deciding how they will divide their funding across different public health services in their area, including vaccination. Each funded vaccine has eligibility criteria. Patients that are not eligible must purchase the vaccine from the pharmacy or their general practitioner.

Since it is potentially the most rapid and convenient method, many people may look online to confirm the availability of services, such as which pharmacies provide vaccination services and which do not. Even with the widely accepted popularity of the internet, some pharmacies may not promote this service online despite offering it. Whilst there are rules as to how products can be promoted, no regulations are stating that pharmacies must promote all of the services they offer. It is at the pharmacy owner's discretion as to what they advertise online.^20^ It is important to confirm statistics and discrepancies in this area, to ensure that pharmacy vaccination services are well publicized as an option within the community. Using information gained from this research, there is the capability to present recommendations to pharmacies regarding how to more effectively promote this essential service. This may include the need for information to be updated promptly, and via a consistent source for public ease of access.

Therefore the purpose of this study was to determine what online information the general public can find with regards to pharmacy vaccination services.

## 2 Methods

Ethics approval for this cross-sectional study was gained from the University of Otago Human Ethics Committee (ref: D20/102).

This study defines a pharmacy's ‘online presence’ as having a website, social media, or Healthpoint account that provides information about the pharmacy. Healthpoint is an online platform in New Zealand that provides up-to-date information about healthcare providers, referral expectations, services offered, and common treatments.^21^ ‘Online vaccination information’ is when one of these online platforms states that the pharmacy administers vaccinations as a pharmacy service.

A list of all community pharmacies in New Zealand was extracted from the License Registry on the Ministry of Health (MOH) website.^22^ The geographic details of the pharmacies were collated into a spreadsheet based on the regional DHBs. An initial web search was carried out, including Healthpoint, official or directly related websites, and the Facebook page(s) of the individual pharmacies and/or banner groups.

A random selection of 75 community pharmacies across six DHBs was later contacted by phone. The six DHBs selected included three DHBs from the North Island (Counties Manukau, Lakes & Hutt Valley) and three from the South Island (Nelson Marlborough, South Canterbury & West Coast). The selection of DHBs was based on geographic factors (North or South Island) and population density (large cities, smaller towns, and remote rural communities). The selection process of the individual pharmacies within each DHB was carried out using an online random number generator. From the randomly generated list, the first fifteen pharmacies from each DHB's list were selected as those to be called. The only exceptions to this are in the case of the West Coast DHB (4 pharmacies) and the South Canterbury DHB (11 pharmacies), where all of the pharmacies in both DHBs were contacted due to small numbers. This process ensured an unbiased selection of pharmacies were to be contacted.

The initial phone script was simplified taking into account how busy pharmacies were expected to be during the time the study was conducted (Alert Level 4, COVID-19). The phone script is presented in Appendix 1.

Data were collated regarding exactly which vaccinations were offered at each pharmacy and compared with previous online research. A discrepancy was recorded if, i) the selection of vaccines online did not match the vaccine selections confirmed when contacted, or ii) the online presence contained no information about vaccines available, but when contacted, it was stated that the pharmacy does offer the vaccination service, or iii) the online presence suggested that the pharmacy offers the vaccination service, but when contacted, it was stated that no such service is offered.

## 3 Results

In New Zealand, there are approximately 1049 pharmacies. There are 802 pharmacies in the North Island (NI) and 247 in the South Island (SI).^22^ Table 1 presents the number and percentages of pharmacies with an online presence and with vaccination information. It is evident that despite a higher percentage of pharmacies having an online presence, only a small number publicized the vaccination information.

**Table 1:** Online presence of pharmacies and vaccination information.

Source of information	Pharmacies having presence n (%)	Pharmacies having vaccination information n (%)	Breakdown of pharmacies having vaccination information⁎	
			North Island n (%)	South Island n (%)
Healthpoint	1033 (98.4)	215 (20.5)	175 (21.8)	40 (16.2)
Website	775 (73.9)	329 (31.3)	250 (31.1)	79 (31.9)
Social media (Facebook)	790 (75.3)	135 (2.8)	92 (11.4)	43 (17.4)

⁎ Percentages are calculated based on the total number of pharmacies and not the number having a presence on the specific platform.

Overall 20.5% of pharmacies in New Zealand had Healthpoint vaccination information for the public to access. A review of the pharmacies' Healthpoint profiles demonstrated that across the DHBs in New Zealand 818 pharmacies did not have any vaccination information and 215 pharmacies (175 pharmacies from NI and 40 pharmacies from SI) had vaccination information. Sixteen pharmacies were not on Healthpoint.

With regards to the websites, 31.4% of pharmacies in New Zealand had vaccination information on their websites for the public to access. Table 1 suggests that 775 pharmacies had a website. From this 446 pharmacies did not have any vaccination information and 329 pharmacies (250 were from NI and 79 were from SI) had vaccination information on their website. A total of 274 pharmacies did not have a website.

Overall, 12.9% of pharmacies in New Zealand had vaccination information on their Facebook page that the public can access. A total of 790 pharmacies had a Facebook page and 259 did not. On the Facebook page, there were 135 pharmacies with vaccination information (92 were from NI and 43 were from SI) and 655 had no such information.

### 3.1 Demographic data of selected DHBs and information discrepancies among randomly selected pharmacies

Table 2 presents the findings of the telephonic survey of the 75 randomly selected pharmacies from six DHBs. There exists some population difference between the six DHBs in the phone contact sample. For example, Counties Manukau DHB has a population of 563,210 with a higher proportion of younger and Pacific Island people compared to the national average.^23^ The population of Lakes DHB is 110,410 that has fewer people in the 20–39 age group, and a much higher proportion of Māori people compared to the national average.^24^ Hutt Valley DHB has a slightly higher proportion of Māori and Pacific people compared to the national average in its population of 149,680 people.^25^ Nelson Marlborough DHB has a population of 150,770 with a higher percentage of elderly people, a lower proportion of Māori (10.6%) and Pacific (1.7%) people than the national average, and also a low proportion of the most deprived people.^26^ The combined population of the two smaller DHBs, South Canterbury and West Coast, is 98,630 which is comprised mostly of older people, compared to the national average.^27^^,^^28^

**Table 2:** Number of pharmacies and information discrepancies.

DHB	No. of pharmacies	No. of randomly selected pharmacies	No. of pharmacies with online vaccination information n (%)	Pharmacies without online vaccination information n (%)	No. of pharmacies with information discrepancies n (%)
Counties Manukau	110	15	8 (53.3)	7 (46.7)	2 (13.3)
Lakes	20	15	4 (26.7)	11 (73.3)	2 (13.3)
Hutt Valley	30	15	4 (26.7)	11 (73.3)	6 (40)
Nelson Marlborough	29	15	8 (53.3)	7 (46.7)	7 (46.7)
South Canterbury & West Coast	15	15	7 (46.7)	8 (53.3)	3 (20)
Total	204	75	31 (41.3)	44 (58.7)	20 (26.7)

Table 2 shows that twenty pharmacies (more than one-fourth of the sample) had discrepancies in their vaccination information between the information available online and that was gained during a follow-up phone call. Of the total twenty pharmacies with information discrepancies, thirteen were associated with a banner group and the rest were independent pharmacies. It was also noticed that from the random sample, 73.4% of the pharmacies that were providing vaccination service were from a banner group and the rest 26.6% were independent pharmacies.

## 4 Discussion

In this era of digital information, social media and online presence are popular tools for pharmacies to access the wider community.^29^ With more pharmacies providing services like vaccinations, this study aimed to determine what online information the general public can find concerning pharmacy vaccination services. Three different platforms were searched (Healthpoint, an official website, and Facebook) all of which are typically easily accessible by the general public. In conducting this research, the authors hope to create positive change in vaccination promotion and encourage pharmacies to fully utilise the benefits that a comprehensive online presence can offer. To the best of the authors' knowledge, this is the first study to examine this topic which restricted the authors' ability to make meaningful comparisons with published literature.

The methodological approach whereby the research team approached the pharmacies as members of the public rather than researchers conducting a survey, is important for several reasons. Firstly, it is the public that pharmacies must target in promoting vaccinations to encourage higher uptake and improved vaccine coverage. This may include unvaccinated people or individuals that wouldn't usually get vaccinated. Secondly, such an approach would result in honest and reliable answers from pharmacists by minimizing the non-response and demand bias. The timing was also important given most of the calls were made during the lockdown because of the COVID-19 pandemic. The less invasive phone calls and a general inquiry assumed that the priority for pharmacies was not to be occupied with research during such unprecedented times, but rather to address community health concerns.

Healthpoint was chosen to explore the pharmacies' online presence as it is one of the main platforms where health providers advertise their services to the public. Pharmacy websites were chosen due to being tailored more to what the individual pharmacy provides, and where many in the public may look to find more information about a business. The pharmacies' Facebook pages were selected as a secondary source where the public may look to find information, following the main websites. In addition, many businesses use this form of social media to promote their services and connect more directly with the public. The overarching reason for the lack of vaccination information across the three online sources is most likely due to vaccination itself being a comparatively recent service in pharmacy, and pharmacies not remembering to update their online resources for the public to access.

Even though it was still a low number, there was a higher proportion of pharmacies with website vaccination information compared to the other two sources (Facebook, Healthpoint). A potential reason for this could be that whilst most pharmacies do have a website, they may prefer this online platform to advertise their services. Social media platforms may be perceived as a less ‘formal’ route of business advertisement, leading to some pharmacy owners choosing to maintain a website over a Facebook (or Healthpoint) page.

However, the ‘website’ category was not specific to a pharmacy's website. Many pharmacies did not have their domain and relied instead on an external platform. For example, Unichem and Life pharmacies, operating under Green Cross Health. The Green Cross Health website was an easily accessible and clear resource when looking for the vaccination information provided.^30^ “Our Health Hawkes Bay” also provided a good indication of the vaccination status for every pharmacy within their DHB.^31^

More than a quarter of the sample had an information discrepancy between the information found online, and information reported when contacted. Potential factors such as not updating websites and lack of staff knowledge may have an underlying role in those discrepancies identified. In addition, there was a higher degree of discrepancies among banner pharmacies, compared with independent pharmacies. An interesting statistic was that 73% of the banner groups offered vaccinations, whereas 26% of independent pharmacies offered this service. A recurrent finding of the research team was that discrepancies occurred more frequently with banner group pharmacies. This can be attributed to the long lists of advertised vaccines leaving pharmacies more susceptible to an error during phone calls.

How frequently a pharmacy updates their service information, and whether the pharmacy is responsible for updating this themselves may also contribute to the results. For example, an independent pharmacy would likely update information on their website or social media personally. The lack of an overarching banner group to assist with and regulate promotion may encourage independent pharmacies to take better advantage of maintaining an up-to-date online presence. When a banner group pharmacy relies on a third party to upload and update information, it is possible for a higher chance of delays or miscommunication to occur. This is supported by the finding that most information discrepancies occurred in banner group pharmacies. The banner groups accounted for in the sample were Green Cross, Countdown, Chemist Warehouse, and Pharmacy 44.

Healthpoint is an independent organisation that pharmacies can use as a platform to advertise information to the public.^21^ This ‘external’ nature of this source may contribute to a delay in providing updated service information. The researchers found some of the Healthpoint vaccine information contradicted the information found when the sample pharmacies were called.

Some common trends that the researchers found regarding the pharmacies that were contacted were that when some pharmacies offered no vaccines, they often recommended nearby pharmacies or medical centres that did offer vaccinations. Some staff members only provided little to no information about the vaccination when asked often due to a lack of knowledge. This lack of knowledge made it difficult to gather information from staff members when vaccinating pharmacists were not available. Some staff members were reluctant to answer questions, preferring to refer to the vaccinating pharmacist instead. Even when staff members were aware of the vaccination service, confusion was sometimes present regarding which vaccines were provided and which were not. The overall discrepancies identified were often regarding the variety of vaccines that pharmacies provided. For example, pharmacies that promoted a wide range of vaccinations online appeared to be more prone to discrepancies occurring, due to an increased likelihood of staff members unaware of the full range, or specific vaccines not in stock.

When asked about the vaccines pharmacies were able to administer, some were available instantly. Most commonly available was the influenza vaccine, with other vaccines reportedly needing to be ordered, such as the whooping cough vaccine. This often differed from what the pharmacy online information suggested; promoting a range of different vaccinations that were seemingly available to receive immediately. The influenza vaccine is reformulated and recommended to be administered annually, with a specific funded eligibility period. Other available vaccines provide coverage across multiple years, and may only reach similar levels of periodic demand during events such as a pandemic. This may be why other vaccines were commonly available for order upon request.

The researchers would like to acknowledge that despite the low numbers of pharmacies throughout NZ providing vaccination information online, there was an increased demand for this service based on the phone calls from the sample of pharmacies. The researchers found that when asking about a pharmacy's vaccination service via phone, appointments commonly needed to be booked, or the pharmacy reported a low supply of vaccines remaining. This may indicate the public's awareness that some pharmacies do administer vaccines, despite the lack of a consistent online source. Despite the study not investigating the public opinions of pharmacies vaccinating, it is clear that demand does exist.

### 4.1 COVID-19 and influenza vaccinations

It is important to highlight the climate in which this research was carried out. The research investigating the online presence and availability of vaccination-related information was initiated in late March 2020, and completed on April 16th 2020. New Zealand moved into the Level 4 COVID-19 lockdown on March 25th, and moved to Level 3 on April 28th 2020. This had an impact on how some services were provided.

Additionally, the 2020 annual influenza programme for eligible patients was initiated earlier than usual, and promotion recommending vaccination as a means of mitigating pressure on the health system led to increased uptake of this vaccine.^11^^,^^32^^,^^33^ With heightened demand for the influenza vaccine and less justification to carry out other vaccinations, it may be logical to anticipate a change in vaccination promotion over this period. An observed trend, with increased numbers of ‘one-off’ social media posts regarding this vaccine was noted, alongside pharmacies reporting on phone calls that the influenza vaccine was the only one available at that time.

The number of pharmacies that provide vaccination services, the public accessibility of this information, and the impact a lack of knowledge may have on immunisation rates were unknown. This study provides a better understanding of how many pharmacies offer the vaccination service, alongside which vaccinations they provide, and how well this is advertised. While there is not one consistent source that the public can refer to for pharmacy vaccination information, fragments of information are spread across multiple platforms, and in some cases do not appear online at all. The research team believes that an up-to-date, comprehensive, freely accessible, and trusted online resource is essential to ensure that pharmacist vaccination remains a valuable and viable service.

It has been acknowledged above that a demand exists for pharmacist vaccination, and that effective utilisation of this service has numerous benefits for immunisation coverage, easing of vaccination burden, and more. Areas for future research include evaluating the public's awareness of pharmacist vaccination as a whole, and determining if the further promotion of vaccination in pharmacies would change where they choose to receive these. There is also the potential to create a new online resource that more effectively promotes this essential service. Additionally, it would also be timely to investigate the impact of COVID-19 on pharmacy vaccination services.

The study has several strengths. Attempts to reduce bias were consistent throughout our study. This study randomly selected a group of pharmacies to be contacted, whilst respective DHBs were intentionally chosen to provide geographic and population-based variation. The pharmacies were all called within the same two-day period, to gather information in the most similar working climate possible.

Additionally, the researchers were able to identify online platforms which require regular updating to ensure that they remain valuable. Drawing attention to areas where customer experience can be improved and information made more readily available aligns closely with the overall aim. In conducting this research, the team hopes to create positive change in vaccination promotion and encourage pharmacies to fully utilise the benefits that a comprehensive online presence can offer.

Finally, the phone call script used to contact the randomly selected pharmacies was well designed and straightforward. The information collected from the phone interviews was written during or straight after the interview to reduce recall bias.

Several limitations to this study are acknowledged. Firstly, human error may have been a factor in the results, as it could not be guaranteed that the staff member contacted was proficient in their knowledge of vaccines offered. Additionally, following the phone script precisely was not always practical in the case of every pharmacy, and some variation may have occurred. Approaching the phone calls as a member of the public made it difficult to consistently discuss vaccinations with the vaccinating pharmacist. This is however true to a real-life situation, and inaccuracies that occurred, as a result, are expanded on in the discussion.

The need to gather information amid the COVID-19 outbreak was a significant consideration to factor into the methodology. Adopting a streamlined approach with a set ‘script’ (refer to appendix) generally enables the rapid gathering of the necessary information, and with the least inconvenience to the pharmacy. It can, however, provide less flexibility for respondents.

Finally, the record of pharmacies obtained from the License Registry (accurate to April 16th, 2020) listed some locations that had permanently closed.^22^ Nevertheless, every effort was made to remove any of these identified pharmacies from the final results.

## 5 Conclusions

Pharmacist vaccination in NZ is a developing service, with the funding status and expansion of vaccinations that pharmacists can administer constantly evolving. With the accessibility of pharmacies throughout NZ and the ability to provide vaccinations, it is important to determine how readily available vaccine information is to the public. Across every online platform researched, low rates of vaccine information were found; 30% on their websites, 20% on Healthpoint, and 13% on social media. A lack of online presence across multiple pharmacies is a pressing issue. NZ pharmacies do not currently have high levels of online presence advertising vaccination services. Updating this information is essential to avoid discrepancies and maintain a reliable service, reduce barriers to access, and ensure public confidence. Increased accessibility to vaccinations leading to better uptake is a concept highlighted throughout the literature surrounding this research. Therefore, improving the amount and quality of this information is pertinent at this time as, in the case of the COVID-19 vaccine, the pharmacy sector will be well placed to conduct vaccinations on a large scale.

## Funding

This research received no specific grant from any funding agency in the public, commercial, or not-for-profit sectors.

## Author contributions

MA and PN made a substantial contribution to the conception and design of the study. All authors contributed to the interpretation of the data. All authors critically revised the manuscript for important intellectual content and approved the final version to be published. All authors agree to be accountable for all aspects of the work in ensuring that questions related to the accuracy or integrity of any part of the work are appropriate investigated and resolved.

## Declaration of Competing Interest

The authors declare that they have no conflicts of interest to disclose.

## Appendix A Supplementary data

Phone call script Supplementary material
