# Supplementary material for: In this digital age, how easily accessible is pharmacist vaccination information? The case of New Zealand
Source: Explor Res Clin Soc Pharm. 2021 Jun 12;2:100033. doi: 10.1016/j.rcsop.2021.100033 (PMC9032484; doi:10.1016/j.rcsop.2021.100033)
Supplement: Supplementary file 1 — Phone call script [file mmc1.docx]

**Appendix 1**

**Phone Call Script**

| Hi there, do you guys offer any vaccines in the pharmacy?  YES - I’m actually making this enquiry on behalf of my (family member). I know that he/she needs an influenza vaccine. He/she also needs another vaccine apparently, but I don’t quite remember the name of the vaccine. Could you please let me know what other vaccines you guys offer in the pharmacy, or is there anything online that I can refer to?  Thanks for your time and have a good day!  NO - No worries, I’m just calling in to check before I enquire about the vaccines. Thanks for your time and have a good day! |
| --- |
